# Differential effects of everyday-life social support on chronic pain

**DOI:** 10.1186/s12883-024-03792-z

**Published:** 2024-08-28

**Authors:** Martin Weiß, Annalena Jachnik, Emilia C. Lampe, Marthe Gründahl, Michael Harnik, Claudia Sommer, Heike L. Rittner, Grit Hein

**Affiliations:** 1 Center of Mental Health, Department of Psychiatry, Psychosomatic and Psychotherapy, Translational Social Neuroscience Unit, University Hospital Würzburg, Margarete-Höppel-Platz 1, 97080 Würzburg, Germany; 2 Department of Psychology I: Clinical Psychology and Psychotherapy, Institute of Psychology, University of Würzburg, Würzburg, Germany; 3 Department of Anaesthesiology, Intensive Care, Emergency and Pain Medicine, Centre for Interdisciplinary Medicine, University Hospital Würzburg, Würzburg, Germany; 4 Department of Anaesthesiology and Pain Medicine, Inselspital, Bern University Hospital, University of Bern, Bern, Switzerland; 5 Department of Neurology, University Hospital Würzburg, Würzburg, Germany

**Keywords:** Social support, Pain, Complex regional pain syndrome, Ecological momentary assessment

## Abstract

**Background:**

Social support is a multidimensional construct encompassing emotional support as well as pain-focused care and attention, also known as solicitous support. One the one hand, social support is widely believed to positively influence pain symptoms, their intensity, and the ability to cope and influence pain. On the other hand, social support can be negative if it conflicts with the patient’s needs or even causes discomfort. How different types of social support influence pain is not very well understood especially because most of the present research originates from laboratory studies, raising uncertainties about its generalizability to the everyday life of individuals with chronic pain.

**Methods:**

Here, we tested the effects of emotional, solicitous, and negative social support on pain intensity cross-sectionally in everyday life. We collected data from 20 patients with acute complex regional pain syndrome using a smartphone-based Ecological Momentary Assessment with up to 30 survey prompts over a period of five consecutive days.

**Results:**

Our results showed that solicitous social support decreased pain, in particular in male patients. Emotional support was beneficial on pain in women but not in men.

**Conclusions:**

Taken together, these findings highlight the differential effects of social support in every-day life on chronic pain.

## Background

Patients suffering from chronic pain show highly individual and heterogeneous patterns of pain intensity and symptoms [1]. Some people are severely affected by chronic pain in their everyday lives, while others cope quite well with it. One factor that might contribute to these individual differences is social support [2]. There is evidence that social support enables the reappraisal of pain, reduces pain-related thoughts, and even lowers the perceived intensity of pain [3]. Additionally, social support correlates with an increase in positive emotions and a lower prevalence of depression [2, 4]. In contrast, the lack of social support is associated with an increase in pain [5, 6] and facilitates pain chronification [7, 8]. This could form a vicious cycle, as disrupted social support patterns reinforce negative cognitive processes or reduce quality of life [9, 10].

Social support can be categorized into different types, such as emotional, solicitous, and negative social support. While emotional support involves an expression of affection and empathy towards the recipient, negative support describes negative reactions of others such as criticizing or ignoring them. Solicitous support involves helping behavior and concern for the recipient [11–13]. These different types of support can have varying effects in the recipient. For example, extensive pain-related concern of a social partner in relation to chronic pain experiences can have a pain-intensifying effect [14–16]. Negative reactions such as frustration with a loved ones’ pain have previously been linked to increased pain and disability in chronic pain patients, whereas emotional support was associated with a decrease in pain [13, 17, 18]. So far, studies comparing the effects of the different types of social support on chronic pain are rare. In addition, most of the current evidence stems from laboratory research, which raises questions about the generalizability of the results in patients’ everyday life.

In the present study, we tested the effects of emotional, negative, and solicitous support on chronic pain intensity in everyday life. Patients suffered from complex regional pain syndrome (CRPS), defined by the presence of pain accompanied by sensory, motor, and autonomic dysfunction in a single extremity [19, 20]. We chose CRPS as part of a large, prospective research project. CRPS is a syndrome that typically occurs after an injury, surgery, or other medical events like strokes [21, 22]. Symptoms include severe, continuous pain and sensitivity in the affected extremity, swelling, changes in temperature and color as well as a significant change in the perception of the affected body part based on the Budapest criteria [23]. Previous research on social support and CRPS is very limited. A study by Feldman and colleagues [24] examined the association between daily CRPS pain, negative mood, and social support in general using daily diary entries, revealing a pain-reducing effect of perceived support. Another study investigating the frequency at which patients with CRPS receive different types of social support pointed to perceived emotional support as the most common source [25]. However, it is unclear how different types of social support affect pain intensity in everyday life. To investigate the effects of support types on CRPS in everyday life, we used Ecological Momentary Assessment (EMA). EMA consisted of a smartphone-based survey in which patients indicated the quantity, type, and quality of their everyday-life social contacts, including the provision of social support, several times a day (see Methods for details). This kind of assessment captures the relationship between different types of social contact and clinical symptoms (in this case, pain) in real time, at high resolution, and in ecologically valid settings [26–35]. Furthermore, EMA helps to reduce recall biases, as it measures current behaviors and experiences rather than retrospective memories [36, 37].

We hypothesized that emotional support has a pain-reducing effect, whereas negative support leads to an increase in subjectively perceived pain. For solicitous support, two alternative hypotheses seem plausible: On the one hand, high levels of this type of support (i.e., too much care) could lead to an increase in momentary pain intensity. This hypothesis is based on the observation that patients report higher pain intensity if their social environment shows excessive attention and concern about their pain syndromes. On the other hand, high levels of solicitous support could also result in a decrease in pain, as patients suffering from chronic pain might benefit from any kind of positive social support.

## Methods

### Participants

Patients were recruited as part of the current cohort of the monocentric *Resolve*PAIN study at the Centre for Interdisciplinary Pain Medicine at the University Hospital Würzburg, and recruitment followed the *Resolve*PAIN study protocol (https://drks.de/ – registration number: DRKS00016790; registration date: 20.02.2019). The individuals in the control group were recruited via an online recruitment portal of the University of Würzburg. The study protocol was approved by the ethics committee of the medical faculty of the University of Würzburg (vote #104/20) and complies with the Declaration of Helsinki. Data collection took place between December 2022 and December 2023. The final sample consisted of 20 patients with CRPS (*M*_age_ = 54.35, *SD* = 11.81; 13 women [65%]) and 16 individuals from the general population in the control group (*M*_age_ = 57.50, *SD* = 11.35; 11 women [69%]). During EMA, participants in the control group completed an average of 28.31 prompts (i.e., 94% compliance, range 25–30) and reported an average of 13 social interactions (range 2–21). Patients responded to an average of 26.45 prompts (i.e., 88% compliance, range 14–30), of which an average of 21.6 included social interactions (range 14–28). While the overall compliance did not differ significantly between the patient and the control group (*p* = .067), patients reported significantly more social interactions than the control group (*p* < .001).

### Participation criteria

General study requirements included sufficient German language skills. For the control group, we excluded individuals with pain disorders, prescription of pain medication, neurological disorders, current neurological treatment, reading problems despite corrected vision, hearing problems despite hearing aids, or fine motor problems. In the patient group, it was ensured that patients currently had CRPS-related pain and that they were able to use a smartphone, hear alarms, and read the questionnaires.

### Procedure

Eligible participants of the patient and control group were invited to the pre-session. After receiving detailed study information, participants’ schedules for the measurement days were individually adjusted to allow a 12-hour measurement time window, starting one hour after the usual wake-up time. Next, all participants provided information about their medication plan as well as sociodemographic data and filled in questionnaires (see below). Finally, each participant practiced completing the EMA survey on the study smartphone in two standardized example situations (social, non-social). After five days of EMA, patients returned the study smartphone by mail and received a survey link to the post-questionnaires, while participants in the control group filled in questionnaires and received financial compensation in a final in-person post-session.

### Apparatus

Trait questionnaires and sociodemographic data were collected at the pre- and post-session using the online survey platform SoSci Survey (https://www.soscisurvey.de). The subjective data from everyday life were collected on a study smartphone using the Android-based app movisensXS (movisens GmbH). All participants were provided with study smartphones equipped with a compatible version of the Android operating system. They were then prompted by acoustic alarms to answer the EMA surveys within their individually adjusted time windows.

### Trait questionnaires

In the pre- and post-session, participants answered the German versions of different personality questionnaires (i.e., time invariant covariates), most of them for exploratory purposes which were not tested here. In the pre-session, we assessed the multidimensional scale of perceived social support (MSPSS; [38]; 12 items on a 7-point Likert scale; α = 0.91), the Lubben social network scale (LSNS-6; [39]; 6 items with scores ranging on a scale from 0 to 5; α = 0.87), the 2-item short form of the pain self-efficacy questionnaire ([40]; 2 items on a 7-point Likert scale; α = 0.98) as well as the loneliness and isolation during social distancing scale ([41]; 25 items on a 5-point Likert scale with five factors; α = 0.57 − 0.89). In the post-session, participants answered the patient health questionnaire-8 (PHQ-8; [42]; 8 items on a 4-point Likert scale; α = 0.90), the generalized anxiety disorder-7 (GAD-7; [43]; 7 items on a 4-point Likert scale; α = 0.91), the anxiety sensitivity index-3 ([44]; 18 items on a 5-point Likert scale; α = 0.93) and a ten-item personality measure ([45]; 10 items on a 7-point Likert scale with two items per Big Five factor; α = 0.53 − 0.72). In addition, the CRPS Severity Score was assessed as a continuous score to indicate the severity of CRPS [46].

### Course of the EMA study

Participants underwent EMA on five consecutive days including a weekend (six prompts per day, max. 30 prompts per person). Participants received these six prompts within a personalized time window of twelve hours each day, starting one hour after the usual wake-up time. The prompts were sent randomly, with at least half an hour between two consecutive prompts. Each prompt was signaled by a 15-second alarm and displayed on the screen of the study smartphone for a total of five minutes. Participants had the option of postponing each prompt twice for five minutes or dismissing it. If participants dismissed the prompt or did not respond to the last postponed prompt, it was counted as unanswered. In each survey, patients first indicated the extent of their current CRPS-related pain (“Please rate the extent of your current pain in the area of your injury/fracture.”) on an 11-point scale from 0 (no pain) to 10 (maximum pain). Participants in the control group were asked about general unspecific pain on the same scale. We then asked all participants how long it had been since their last social interaction. For social interactions ≤ 30 min ago, participants were presented with the social interaction questionnaire. Otherwise, they were required to answer a similarly structured activity questionnaire to prevent avoidance behavior due to time savings (see also [29, 35]). In the social interaction questionnaire, participants were asked about the characteristics of their latest social interaction: its duration, nature (face-to-face, virtual, written), the number of interaction partners, their gender, and the degree of familiarity with the main interaction partner. In line with previous studies [13, 47, 48], the items examining solicitous support included whether the interaction partner performed chores for the participant, brought them food/drinks, or asked them to rest. Emotional support was assessed by asking for affection, attention, and empathy of the interaction partner. Finally, negative support was measured by the extent of an irritated, frustrated reaction, or being ignored by the interaction partner.

### Statistical analyses

First, we tested for differences between the patient and the control group using two-sample *t*-tests. As the pain ratings in the control group were low on average (mean = 1.1), had a low variance (*SD* = 0.4), and were highly skewed (kurtosis = 2.43), we excluded the control group from the main analysis. We analyzed our hypotheses on the patients with CRPS using a linear mixed effects model with the lme4 package [49] in R Studio. A recent study highlighted the benefit of using mixed models to analyze the variability of pain in EMA as they provide higher statistical power and greater sensitivity [50]. *P*-values were obtained via the Satterthwaite approximation of degrees of freedom [51]. In the current study, we did not explicitly focus on gender effects. However, as gender is an important aspect of social interaction [29, 35, 52], we included a binary gender variable that encoded female vs. male patients. All within-person predictor variables were person-mean centered and all between-person variables were group-mean centered to facilitate their interpretation [53, 54]. Due to the extreme skewness of negative social support (NSS; 2.73 in the control group and 3.88 in the CRPS group), we did not include NSS into our model, although this was initially planned. The first model included CRPS-related pain as the outcome variable, the two support types – emotional social support (ESS) and solicitous social support (SSS) – and gender as main effects, the interactions gender x ESS and gender x SSS, and a random intercept per participant. In the second model, we updated the first model by adding depression (PHQ) and anxiety (GAD) as main effects to account for individual mental health conditions. To evaluate the stability of any significant main effect of ESS and/or SSS on CRPS pain, we conducted a bootstrap sensitivity analysis using 1000 resamples from the original dataset. This analysis focused on the respective coefficient to assess its influence on pain across varied samples. The alpha level for significant results is α = 0.05.

## Results

### Confirmed differences between patient and control group

Patients with CRPS had an average CRPS Severity Score of 12.6 (*SD* = 2.23, range = 9–16). The duration between diagnosis and inclusion in the cohort of the monocentric study was 72.25 days (range 7 to 229 days), with EMA occurring on average 229.35 days after diagnosis. Patients with CRPS and healthy controls were roughly the same age (*p* = .422). As expected, pain (*p* < .001, *d* = 1.55) as well as mental health impairments such as depression and anxiety were elevated in patients with CRPS (*p*s < 0.001, *d*s 1.62 and 1.64, respectively; see Table 1). As expected, patients with CRPS received more solicitous support compared to the control group (*p* = .029, *d* = 0.765). However, patients and controls did not differ significantly in their general social support (networks) according to MSPSS (*p* = .055) and LSNS (*p* = .730).

**Table 1:** Descriptive statistics and comparisons between the patients with CRPS and the control group

	patients with CRPS (*N* = 20)	control group (*N* = 16)		
	Mean ± *SD*	Mean ± *SD*	*p*	Cohen’s *d*
Age	54.35 ± 11.81	57.50 ± 11.35	0.422	0.272
PHQ*	8.94 ± 5.47	2.00 ± 2.65	< 0.001	1.62
GAD*	7.11 ± 5.12	0.93 ± 1.49	< 0.001	1.64
MSPSS	62.75 ± 10.76	69.06 ± 8.29	0.055	0.657
LSNS	16.50 ± 5.39	17.12 ± 5.33	0.730	0.117
SSS	2.02 ± 1.34	1.02 ± 1.29	0.029	0.765
ESS	2.67 ± 1.63	2.88 ± 1.30	0.674	0.141
NSS	0.26 ± 0.45	0.27 ± 0.40	0.938	0.026
Pain	4.02 ± 2.62	1.10 ± 0.40	< 0.001	1.55

Note. CRPS = complex regional pain syndrome; ESS = emotional social support; GAD = generalized anxiety disorder-7; LSNS = Lubben social network scale; MSPSS = multidimensional scale of perceived social support; NSS = negative social support; PHQ = patient health questionnaire-8; SSS = solicitous social support. *p* indicates the significance according to a two-sample *t*-test. * Data was only available for 18 patients with CRPS as two did not fill in the post-survey, in which PHQ and GAD were assessed

### Gender differences in emotional and solicitous support

**Fig. 1:**
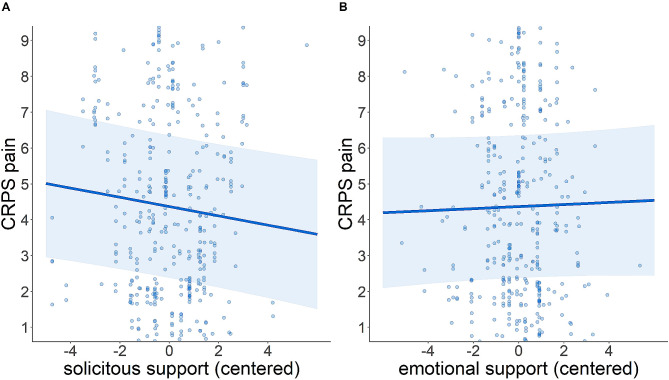
(**A**) Significant main effect (predicted values) of solicitous support on CRPS pain (numerical rating scale). (**B**) Non-significant main effect (predicted values) of emotional support on CRPS pain (numerical rating scale). The shaded area indicates confidence intervals

**Fig. 2:**
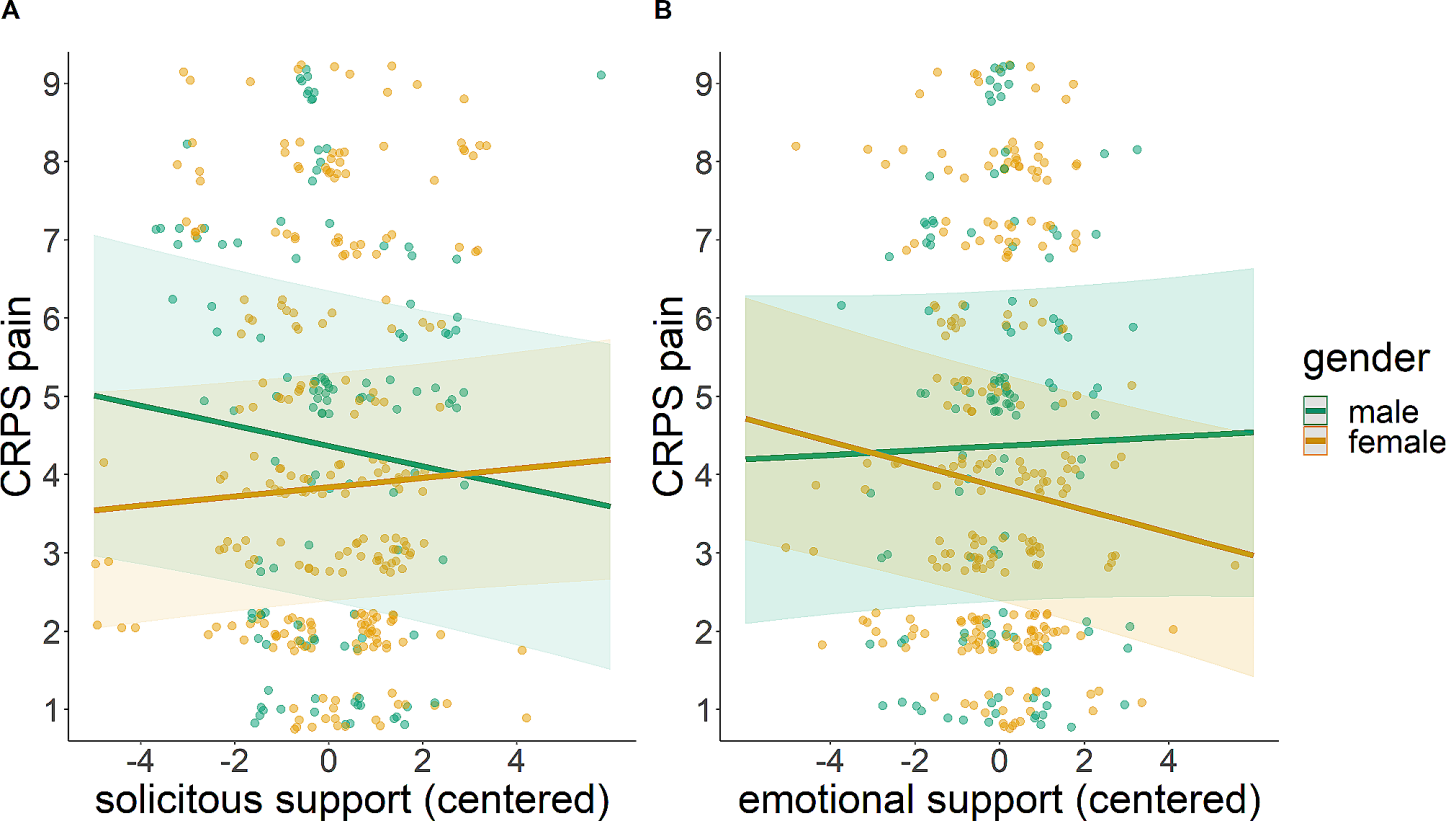
(**A**) Interaction effect between solicitous support and gender on CRPS pain (numerical rating scale). (**B**) Interaction effect between emotional support and gender in CRPS pain (numerical rating scale). The shaded area indicates confidence intervals

The results of the linear mixed effects model are summarized in Table 2. The model addressing within-EMA effects (M1) showed a main effect of SSS (*B* = − 0.13, 95% confidence interval [CI] = [-0.23, − 0.02], *p* = .016), indicating decreasing CRPS-pain with higher SSS as illustrated in Fig. 1a. Supporting this indication, the bootstrap results provided a 95% confidence interval for this effect (-0.209, -0.049), which does not include zero and remains negative. This demonstrates a statistically significant and robust negative relationship between SSS and CRPS pain: when an individual had a social interaction with higher SSS, they reported decreased pain. Gender (*B* = − 0.53, CI = [-2.99, 1.93], *p* = .675) and ESS (*B* = 0.03, CI = [-0.09, 0.14], *p* = .624; Fig. 1b) had no significant main effects. However, we detected significant interactions between gender and SSS (*B* = 0.19, CI = [0.06, 0.32], *p* = .006; Fig. 2a) as well as gender and ESS (*B* = − 0.17, CI = [-0.32, − 0.03], *p* = .019; Fig. 2b). Women reported higher CRPS-pain with increasing SSS compared to men. Simple slopes analysis revealed that the slope was significant for men (*p* = .02), but not for women (*p* = .16), suggesting that the pain-decreasing effect of SSS may be limited to men. In contrast, women reported lower CRPS-pain with increasing ESS compared to men. In this case, simple slopes analysis revealed that the slope was significant for women (*p* < .001), but not for men (*p* = .62). In the model with added mental health conditions (M2), higher depression scores were associated with higher pain (*B* = 0.29, CI = [0.04, 0.54], *p* = .021). Anxiety showed no significant effect (*B* = 0.08, CI = [-0.21, 0.38], *p* = .572). The fixed effects part (marginal *R*²) of the mental health model explained 45.8% of variance, whereas the EMA model explained only 1.2% of variance. However, the conditional R² (including random effects) was comparable (88% in the EMA model and 88.5% in the mental health model).

**Table 2:** Results of the linear mixed effects models including a model with Ecological Momentary Assessment variables and a model containing additional mental health factors (M2)

	EMA model (M1)				M1 + mental health (M2)		
Predictors	B	CI	*p*		B	CI	*p*
**(Intercept)**	**4.37**	**2.38**,** 6.35**	**< 0.001**		**3.01**	**1.26**,** 4.76**	**0.001**
gender [female vs. male]	-0.53	-2.99, 1.93	0.675		-0.17	-2.28, 1.94	0.876
**SSS**	**-0.13**	**-0.23**,** -0.02**	**0.016**		**-0.13**	**-0.23**,** -0.03**	**0.015**
ESS	0.03	-0.09, 0.14	0.624		0.03	-0.09, 0.14	0.621
**gender × SSS**	**0.19**	**0.06**,** 0.32**	**0.006**		**0.25**	**0.11**,** 0.38**	**< 0.001**
**gender × ESS**	**-0.17**	**-0.32**,** -0.03**	**0.019**		**-0.18**	**-0.33**,** -0.04**	**0.015**
**PHQ**					**0.29**	**0.04**,** 0.54**	**0.021**
GAD					0.08	-0.21, 0.38	0.572
ICC	0.88				0.79		
*N*	20				18		
Observations	430				381		
Marginal *R*^2^/Conditional *R*^2^	0.012 / 0.880				0.458 / 0.885		

Note. ESS = emotional social support; GAD = generalized anxiety disorder-7; ICC = intraclass correlation; M1 = model 1; M2 = model 2; PHQ = patient health questionnaire-8; SSS = solicitous social support. Bold letters and numbers indicate significant effects

## Discussion

Assessing the daily experiences of patients with chronic pain conditions is crucial for a better comprehension of the disease and for identifying characteristics of social support that could enhance pain treatment strategies. In the present EMA study, we contributed to understanding the effects of emotional and solicitous social support on daily pain experiences of patients suffering from CRPS. The results of this cross-sectional study showed a positive, pain-reducing association between solicitous support and pain. The gender of the patient interacted with both types of social support: With increasing levels of solicitous support, men reported decreasing pain. In contrast, increasing levels of emotional support were related to decreasing pain in women (Fig. 2).

Previous research has suggested a U-shaped function of social support on pain: it has beneficial effects up to a certain threshold, beyond which it results in a pain-enhancing focus on the disease-related burden and disability [55]. In the current study, we demonstrated that a decrease in pain ratings was linked to higher perceived solicitous support during social interactions. This finding contrasts with other research, where an increase in pain intensity was observed with increasing solicitous support (e.g., [14, 15, 17, 18]). The discrepancy in findings could result from differences in pain conditions (e.g., chronic pain vs. acute pain) and study settings (real life vs. laboratory). Moreover, solicitous support is assessed differently in different studies. In our measurement of solicitous support, the patient reported on the behavior of the interaction partner (taking over chores, asking to rest, bringing food/drinks), without indicating its appropriateness. In other studies, solicitous support is measured based on the reaction of the patient (e.g., being pleased or refusing help), showing that being offered unwanted help can increase pain (for review, see [48]). Future studies should therefore investigate whether the support in question was accepted, rejected, or even ignored.

In contrast to previous studies [56, 57], we found no main effect of emotional support on pain intensity of patients with CRPS. However, in these studies, the target population were tumor patients and women undergoing mammography screening, a completely different population from CRPS. Nevertheless, this discrepancy could be explained by patients’ preferences for pain-related support being influenced by the acuteness of their condition and current treatment status, such as the degree of independence, acute lifestyle changes, and the implementation of new self-management strategies. These stages may determine whether solicitous or emotional support is more valuable at a given point in time [58], which underlines their context-dependence. Furthermore, we found that reduced pain was associated with higher emotional support in female patients. This finding is in line with previous studies showing a beneficial effect of emotional support on women with regard to depression [4] and pain [59]. In more detail, Samulowitz and colleagues showed that strong emotional support in women (with frequent pain) predicted less frequent pain at follow-up. In contrast to female patients, there was no significant effect of emotional support in male patients. One possible explanation might be that male patients are less likely to communicate pain-related emotions than female patients [60] which in turn might trigger less emotional support from their interaction partners.

Regarding solicitous support, research on gender differences is limited. In one study, the authors used a questionnaire to categorize spouses as high or low in solicitous support [61]. The results showed that for both women and men, perceived spousal solicitousness was associated with greater clinical pain. Only in men, higher spousal solicitous support correlated with greater self-reported disability (i.e., the extend to which functional daily activities are restricted by pain), while women with high solicitous support showed lower pain tolerance, poorer performance on functional measures (e.g., longer walking time for certain distance), and higher opioid use. These findings indicate gender-specific effects of spousal responses on pain adjustment, but in a different way than in our data. While Fillingim and colleagues used one time point to classify partners’ solicitous support, we quantified solicitous support in each interaction (and with different interaction partners). Therefore, our results might differ due to situation-dependent social and gender-specific role expectations that shape pain patients’ responses to different types of social support and pain management. While women might be more inclined to seek emotional support and use emotion-focused coping behaviors, men might tend to apply approaches consistent with a preference for solicitous support [59, 62, 63].

The observation of gender-specific effects is in line with previous evidence. However, in our study, they might have been biased by an imbalanced sample with more female than male participants. Sensitivity analysis on the main effect of solicitous support showed that the overall sample size was sufficient to test our main hypotheses. Nevertheless, a larger and gender-balanced sample would be desirable to test the stability of our results. To minimize the burden on patients, we opted for a moderate duration of the EMA study. While this approach facilitated participation, it potentially restricted our ability to thoroughly explore the dynamics of social contact across a variety of interaction partners. For a more comprehensive examination of a wider range of interactions (both higher in quantity and diversity), an extended sampling period would be advantageous. Furthermore, comparing our results with data from patients suffering from other chronic pain conditions and/or from other cultures and regions could provide insight on whether the effects of social support in chronic pain conditions are generalizable or whether they are disease-specific.

## Conclusion

Our study provides novel insights into the relations between different types of everyday-life social support and pain intensity in patients with CRPS. We provide evidence for an overall positive effect of solicitous support, which seems to be stronger in male patients. In contrast, there is evidence that female patients with CRPS benefitted more from emotional support. These results highlight the importance of social modulation processes in CRPS and can inform psychological pain interventions.

## Data Availability

The datasets generated and/or analyzed in the current study are not publicly available due to the sensitive nature of patient data. However, they can be obtained from the corresponding author upon reasonable request.
